# Does a dose-response relation exist between spinal pain and temporomandibular disorders?

**DOI:** 10.1186/1471-2474-10-28

**Published:** 2009-03-02

**Authors:** Birgitta Wiesinger, Hans Malker, Erling Englund, Anders Wänman

**Affiliations:** 1 Department of Research and Development, Sundsvall Hospital, 85186 Sundsvall, Sweden; 2 Department of Clinical Oral Physiology, Faculty of Medicine, Umeå University, Umeå, Sweden

## Abstract

**Background:**

The aim of this study was to test whether a reciprocal dose-response relation exists between frequency/severity of spinal pain and temporomandibular disorders (TMD).

**Methods:**

A total of 616 subjects with varying severity of spinal pain or no spinal pain completed a questionnaire focusing on symptoms in the jaw, head and spinal region. A subset of the population (n = 266) were sampled regardless of presence or absence of spinal pain. We used two different designs, one with frequency/severity of spinal pain, and the other, with frequency/severity of TMD symptoms as independent variable. All 616 participants were allocated to four groups, one control group without spinal pain and three spinal pain groups. The subjects in the subset were allocated to one control group without TMD symptoms and three TMD groups. Odds ratios (ORs) were calculated for presence of frequent TMD symptoms in the separate spinal pain groups as well as for frequent spinal pain in the separate TMD groups.

**Results:**

The analysis showed increasing ORs for TMD with increasing frequency/severity of spinal pain. We also found increasing ORs for spinal pain with increasing frequency/severity of TMD symptoms.

**Conclusion:**

This study shows a reciprocal dose-response-like relationship between spinal pain and TMD. The results indicate that these two conditions may share common risk factors or that they may influence each other. Studies on the temporal sequence between spinal pain and TMD are warranted.

## Background

Temporomandibular disorders (TMD) are musculoskeletal pain conditions characterised by pain and dysfunction in the jaw-face muscles and/or the temporomandibular joint. Musculoskeletal pain conditions occurring at various locations may share pathophysiological mechanisms [1-3]. Co-morbidity between TMD, headaches and neck/shoulder pain has been reported in TMD patient samples as well as in samples drawn from the general population [4-10]. Low back pain, one of the most common pain conditions in humans [11,12], has been associated with other pains such as neck pain and headaches [2,13-15], which has been interpreted as a tendency for symptoms to cluster in some individuals [16]. The source of these patterns is not known, but neurobiological sensitization processes [17-19], genetically determined vulnerability and psychological factors [20-22] are commonly given as possible explanations. Results of a 3-year prospective study showed a significantly increased risk of developing a new pain condition with presence of a pain condition at baseline [23]. A more recent prospective study based on patients with non-painful TMD indicated a dose-response relationship between the number of pain sites at baseline (head, back, chest, stomach) and the risk of onset of dysfunctional TMD pain among women [24]. Frequency of headaches was found to have a dose-response relationship with occurrence of musculoskeletal symptoms (e.g. pain in neck, shoulders and low back) in a Norwegian population [7].

We have recently shown that patients with long-term spinal pain (neck, shoulder and/or low back) significantly more often have signs and symptoms of TMD than do matched controls [25]. The associations remained statistically significant also after exclusion of those who reported jaw pain. It is not known whether co-morbidity between TMD and spinal pain occurs within the whole range of variation in symptom frequency and severity. Most analyses in this field have involved dichotomized samples, not taking variations of symptom severity into consideration. The aim of the present study was to test whether a reciprocal dose-response relation exists between frequency/severity of spinal pain and temporomandibular disorders (TMD). We tested the following null hypotheses:

1. Occurrence of frequent TMD symptoms and headaches does not differ significantly between study groups with varying **frequency/severity of spinal pain**.

2. Presence of frequent spinal pain does not differ significantly between study groups with varying **frequency/severity of TMD symptoms**.

## Methods

### Study population

The study population was originally sampled for a case-control study [25]. The subjects were recruited from among employees at four companies and patients at a vocational rehabilitation center. At two of the companies our primary interest was to identify subjects without spinal pain, and at the two remaining companies the employees participated regardless of presence or absence of spinal pain. In total 516 employees completed the questionnaire. One hundred patients with spinal pain were recruited at the rehabilitation center. The study population thus comprised 323 men and 293 women (n = 616) with varying frequency/severity of spinal pain or no spinal pain (Table 1). The study was approved by the Ethics Committee at Umeå University, and all subjects gave their informed consent to participate.

**Table 1:** Characteristics of the study population, with spinal pain as independent variable

	SP-0	SP-1	SP-2	SP-3
Number, men/women	127/128	54/53	93/61	49/51

Mean age, men/women (years)	40.4/42.9	41.3/37.6	38.9/38.7	41.4/39.4

Age range, men/women (years)	20–64/23–62	25–65/26–60	20–59/23–59	25–56/24–61

Intensity of neck pain		3.4	5.2	6.5

Intensity of shoulder pain		3.2	5.2	6.6

Intensity of low back pain		3.7	5.3	6.6

Impact of neck/shoulder pain on ADL		2.1	3.7	6.0

Impact of low back pain on ADL		3.0	4.3	6.7

The Table shows the number of subjects, as well as gender, mean age and age range in the study groups. Mean values (assessed on 11-point numerical rating scales) of pain intensity in the spinal region, and pain interference with activities of daily living (ADL) are shown. Mean values are calculated among those who reported the specific pain location. SP-0 = without spinal pain; SP-1 = with mild spinal pain; SP-2 = with moderate spinal pain; and SP-3 = with disabling spinal pain and attending a vocational rehabilitation programme.

### Assessment of symptoms

The operational definition of 'spinal pain' was pain in the neck, shoulders and/or low back. Symptoms in the jaw-face region, head, neck, shoulder and low back regions were assessed by questionnaire. Presence of symptoms was stated for frequency (never; not now, but previously; once or twice a month; once or twice a week; several times a week; daily), duration (< 1 month; 1 month–1 year; 1–5 years; > 5 years) and intensity. The subjects were also asked to estimate the impact of jaw symptoms, headaches, neck-shoulder pain and low back pain on activities of daily living (ADL). Intensity and ADL was assessed using the 11-point Numerical Rating Scale (NRS) [26].

Presence and severity of TMD was evaluated for the separate symptoms and according to the Helkimo Anamnestic dysfunction Index [27]. This classification grades the severity of symptoms in the jaw-face region into mild (i.e. temporomandibular joint (TMJ) sounds during opening and closing of the jaw and/or tiredness/stiffness in the jaws) or severe (i.e. pain, TMJ locking and/or difficulties in opening the mouth wide).

### Spinal pain as independent variable

With spinal pain as independent variable we tested the hypothesis that occurrence of frequent TMD symptoms and headaches would increase with increasing frequency/severity of spinal pain. A total of 616 subjects were classified into four groups according to the frequency/severity of their reported spinal pain and the related disability. One group comprised subjects without any spinal pain (SP-0) and were therefore designated the control group. The inclusion criterion for those considered to have infrequent spinal pain (SP-1) was spinal pain once or twice a month, at the most. The inclusion criterion for subjects with frequent spinal pain (SP-2) was spinal pain (weekly to daily) that had been present for at least 1 month. Exclusion criteria for subjects in groups SP-0, SP-1 and SP-2 were current sick leave or disability pension. The inclusion criterion for patients with disabling spinal pain (SP-3) was frequent, long-term spinal pain and referral to a vocational rehabilitation center for rehabilitation. These subjects had been on part-time or full-time sick leave prior to rehabilitation, owing to spinal pain. The male/female ratio, mean age and age range, mean intensity of pain (assessed on an 11-point NRS) in the neck, shoulder and low back, and the symptoms' impact on ADL (assessed using the 11-point NRS), for the separate groups are presented in Table 1.

### Symptoms of temporomandibular disorders as independent variable

A subset of the population (n = 266) were sampled regardless of reported presence or absence of spinal pain. To test the hypothesis that the occurrence of frequent spinal pain increases with increasing frequency/severity of TMD symptoms, we used this subset of the total study population. The subjects were allocated to four groups based on frequency and severity of TMD symptoms. One group comprised subjects without symptoms of TMD (TMD-0). The inclusion criteria for TMD-1 were infrequently occurring (once or twice a month, at the most) TMD symptoms. The inclusion criteria for TMD-2 were frequent and mild symptoms (TMJ sounds and/or fatigue/stiffness) occurring once a week or more often. The inclusion criteria for TMD-3 were frequent and severe symptoms in the jaw-face region (pain, difficulties in opening the jaw wide, and/or TMJ locking) occurring at a frequency of once a week or more often. See Table 2 for characteristics of the subgroups.

**Table 2:** Characteristics of the subset population, with symptoms of temporomandibular disorders (TMD) as independent variable

	TMD-0	TMD-1	TMD-2	TMD-3
Number, men/women	89/48	23/21	35/28	12/10

Mean age, men/women (years)	38.5/36.0	37.1/32.3	35.3/35.4	36.7/34.4

Age range, men/women (years)	20–65/23–58	26–58/26–43	20–59/23–56	29–58/31–52

Impact of TMD symptoms on ADL		0.7	1.1	3.0

The Table gives the number of subjects, as well as gender, mean age and age range in the study groups. Mean values (assessed using the 11-point numerical rating scale) of the impact of TMD symptoms on activities of daily living (ADL) are shown. TMD-0 = without symptoms of TMD; TMD-1 = with infrequent symptoms of TMD; TMD-2 = with frequent, mild symptoms of TMD; and TMD-3 = with frequent, severe TMD symptoms.

### Statistical analyses

The data analysis was performed in SPSS, version 14.0. Data are presented as prevalence of symptoms in the separate groups. Odds ratios (ORs) and 95% confidence intervals (CIs) were calculated with binary logistic regression analysis. In the analyses we adjusted for age and sex since both factors may relate to spinal pain [28,29] and TMD [30,31]. Results were considered statistically significant if the 95% CI did not include 1. The case groups were compared with the control group. The control group was defined as the group of subjects without spinal pain (SP-0) or without symptoms of TMD (TMD-0). To test the trends for dose-response associations we used Cochran-Armitage Test for Trend [32] with syntax for SPSS (Garcia-Granero, M: ).

## Results

### Spinal pain as independent variable

**Figure 1:**
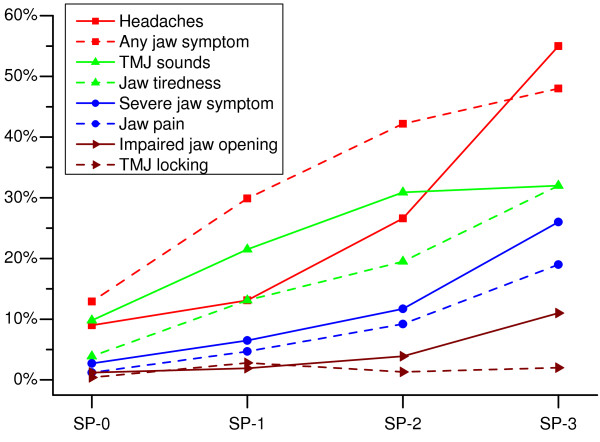
**Spinal pain as independent variable: Prevalence of TMD symptoms and headaches**. Prevalence of frequent symptoms in the jaw-face region and headaches among subjects without spinal pain (SP-0), SP-1 subjects with infrequent spinal pain, SP-2 subjects with frequent spinal pain and SP-3 subjects with disabling spinal pain and attending a vocational rehabilitation programme.

**Figure 2:**
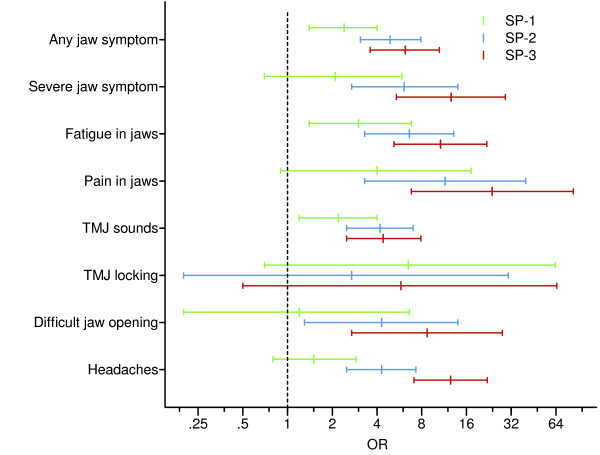
**Spinal pain as independent variable: Odds ratios and 95% confidence intervals for TMD symptoms and headaches**. Odds ratios (ORs) and 95% confidence intervals (CIs) for presence of frequent symptoms in the jaw-face region and headaches among subjects with infrequent spinal pain (SP-1), subjects with frequent spinal pain (SP-2) and patients with disabling spinal pain attending a rehabilitation programme (SP-3), compared with controls (SP-0). ORs and 95% CIs were calculated with binary logistic regression analysis, adjusting for age and sex.

The prevalence of TMD symptoms and headaches in the different spinal pain groups is presented in Figure 1; the ORs and 95% CIs, in Figure 2. The prevalence of fatigue/stiffness, pain, impaired jaw opening, and headaches, as well as the overall prevalence of any TMD symptoms and severe TMD symptoms increased in a dose-response pattern in relation to frequency/severity of spinal pain. The test for trends showed significant (*P *< 0.001) dose-response relations between spinal pain and all TMD variables, except TMJ locking, as well as between spinal pain and headaches.

### Symptoms of temporomandibular disorders as independent variable

**Figure 3:**
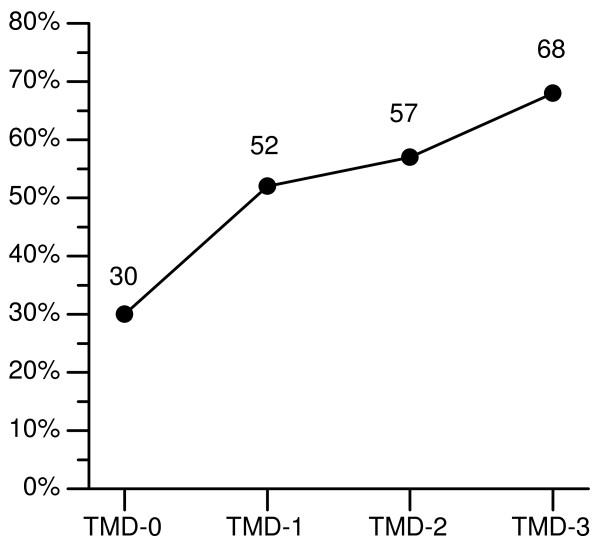
**Symptoms of temporomandibular disorders as independent variable: Prevalence of spinal pain**. Prevalence of frequent spinal pain among subjects without symptoms of temporomandibular disorders (TMD-0), TMD-1 subjects with infrequent symptoms of TMD, TMD-2 subjects with frequent, mild symptoms of TMD, and TMD-3 subjects with frequent, severe symptoms of TMD.

The occurrence of frequent spinal pain increased in a dose-response pattern with increasing frequency/severity of symptoms of TMD (Fig. 3), from 30% in TMD-0 to 68% in TMD-3. The OR for frequent spinal pain increased from 2.8 (95% CI: 1.4–5.7) among those with infrequent TMD symptoms, to 3.3 (95% CI: 1.8–6.2) among those with frequent, mild symptoms, and 5.1 (95% CI: 1.9–13.4) among those with frequent, severe TMD symptoms, compared with the controls. The test for trends showed a significant (*P *< 0.001) dose-response relation between symptoms of TMD and spinal pain.

## Discussion

The present study showed a dose-response relation between frequency/severity of spinal pain and temporomandibular disorders (TMD). The pattern was evident in both directions, the prevalence of frequent TMD symptoms and headaches increasing with increasing frequency/severity of spinal pain, and the prevalence of frequent spinal pain increasing with increasing frequency/severity of TMD symptoms. The test for trends showed significant dose-response associations in both directions. The two tested null hypotheses were therefore rejected.

We have previously shown that patients with long-term spinal pain have a sevenfold risk of reporting pain and dysfunction in the jaw-face region and a fivefold risk of having clinical signs of TMD, compared with matched controls [25]. This finding was recently supported in a cross-sectional analysis based on almost 30,000 adults in the USA [12], indicating a strong relationship between reported spinal pain and jaw-face pain (adjusted OR: 11.3, 95% CI: 9.4–13.5). The present study shows a stepwise positive correlation between severity of spinal pain and pain and dysfunction in the jaw-face region. This dose-response-like pattern should not be interpreted as a sign of exposure and outcome. However, it strengthens previous results of an association between TMD and spinal pain and may point to common underlying biological or psychological mechanisms. It should be emphasized that the results are derived from a cross-sectional study and do not show causality. Owing to the study design we have no information about the temporal sequence of the examined disorders, an essential element in assessing causality. Studies with a prospective design have indicated that presence of a pain condition increases the risk of contracting TMD pain [23,24]. In a recent prospective study the risk for onset of facial pain, meeting research diagnostic criteria for TMD, was almost four times higher among adolescents with back pain at baseline, than among those without back pain [33]. Papageorgiou et al. followed a cohort without low back pain at baseline and noted that musculoskeletal pain at other sites predicted future episodes of low back pain [34]. These results are interesting, but so far there is no sufficient evidence to conclude that back pain precedes TMD, or vice versa. Psychological factors are often co-morbid with chronic pain conditions [21,35]. The temporal sequence of pain and depression is however not clear. In a review addressing this question the majority of studies indicated that depression was a consequence rather than an antecedent of pain [36]. Longitudinal studies on these issues are therefore warranted.

It has been suggested that generalized pain (i.e. fibromyalgia) is at one end of a continuum [37-39]. Vierck presents temporomandibular pain as an example of a focal pain condition where the nociceptive sensory input may contribute to development of generalized hypersensitivity and related susceptibility to further load [40]. In line with this hypothesis one experimental study reports signs of mechanical allodynia in the hindpaw following nociceptive stimuli applied to the masseter muscle of rats [41]. Other experimental studies have shown that perceived muscle pain intensity and distribution is influenced by the stimulation rate (temporal summation) and the number of stimulated afferents (spatial summation) [42]. Temporal summation has been shown in TMD patients, as well as in other chronic pain conditions, suggesting a generalized hyperexcitability of the central nociceptive system [3]. In a large population sample grouped with respect to frequency of reported headaches (< 7 days/month; 7–14 days/month; > 14 days/month) a dose-response pattern was demonstrated between headache frequency and 1-year prevalence of musculoskeletal symptoms (with locations including neck, shoulders, elbows, wrist/hands, chest/abdomen, upper back, low back, hips, knees, ankles/feet) [7]. The contribution of input from the craniofacial nervous system in spreading pain may therefore be of significance and more experimental and clinical studies are needed.

Recent studies have shown that genetic polymorphism, with influence on the metabolism of catecholamines, is highly associated with pain sensitivity and the risk for developing TMD [43-45]. Central sensitization may be one possible explanation for co-morbidity between pain conditions at different locations, as well as presence of allodynia and hyperalgesia [17,40]. Reflex connections between nociceptors and the fusimotor-muscle spindle system may also be involved in the pathophysiologic mechanisms related to pain and dysfunction [46,47].

The allocation of subjects in the present study to different spinal pain groups was based on the participants' reports of pain frequency in the questionnaire. For example, if a subject reported daily shoulder pain, but infrequent low back pain, the grouping was done according to the frequency of shoulder pain. Subjects who had been referred to a rehabilitation programme and who were on sick leave (SP-3) were considered to have more severe spinal pain than subjects with frequent pain but not on sick leave (SP-2). Symptom description in self-report questionnaires may be a limitation in a strict dose-response discussion; however, frequency as well as intensity and duration of pain and dysfunction are important variables in health care seeking behaviour [48,49]. Similarly, in this study, pain severity in the separate spinal pain groups demonstrates stepwise increased mean values of reported pain intensity and impact on ADL (Table 1). In the sub-sample test with symptoms of TMD as independent variable, we included none of the patients from the rehabilitation center. The severity of the TMD symptoms is reflected by the reported interference of jaw symptoms with daily living (Table 2). The formation of groups, aiming at discrete severity categories (dose), therefore seems valid also with regard to the mean intensity level and the impact of the symptoms on daily living.

## Conclusion

The study shows a reciprocal positive dose-response pattern between frequency/severity of spinal pain and temporomandibular disorders. The results indicate a strong co-morbidity between these two conditions, suggesting that they may share risk factors or that they may influence each other. We agree with the recently advocated view of a need for hypothesis-based studies on specific pain-pain co-morbidities [50], but also on pain-dysfunction co-morbidities. The present results are of significance for physicians and dentists, both of whom are expected to manage patients with pain and dysfunction. Collaboration as well as a costing system for cooperation in the diagnosis and management of the two conditions is warranted. Researchers of pain conditions should include the jaw-face region in their efforts to comprehend the pain patient's case history.

## Competing interests

The authors declare that they have no competing interests.

## Authors' contributions

BW was the main author of the manuscript and participated in all stages throughout the work. HM was involved in the study design, interpretation of data and made critical comments on the manuscript. EE performed the statistical analysis and participated in the interpretation of data. AW acted as a supervisor and was involved in all parts of the study. All authors read and approved the final version of this paper.

## Pre-publication history

The pre-publication history for this paper can be accessed here:


